# Comparison of Biological Activities of *Inonotus hispidus* Polysaccharides by Solid-State and Submerged Cultures

**DOI:** 10.3390/ijms27156724

**Published:** 2026-07-28

**Authors:** Yu Wang, Hao Guo, Gen Xu, Xiaobei Li, Ziyin Xu, Zhonghai Li

**Affiliations:** State Key Laboratory of Biobased Material and Green Papermaking, Shandong Provincial Key Laboratory of Microbial Engineering, School of Bioengineering, Qilu University of Technology, Shandong Academy of Sciences, Jinan 250353, China; 15149017570@163.com (Y.W.); m17854118346@163.com (H.G.); xugen2019@163.com (G.X.); lixiaobei722@163.com (X.L.); xzy20011232022@163.com (Z.X.)

**Keywords:** polysaccharide, *Inonotus hispidus*, antioxidant, anti-inflammatory

## Abstract

*Inonotus hispidus* is a medicinal mushroom that produces polysaccharides with potential biological activities. In this study, polysaccharides obtained from fruiting bodies (IH-F) and submerged fermentation broth (IH-L) of *I. hispidus* LQ-1 were compared. IH-F was composed of D-galactose (36.07%), L-fucose (17.22%), D-mannose (15.48%), D-glucose (9.78%), D-arabinose (9.43%), D-xylose (7.29%), glucuronic acid (3.32%), D-galacturonic acid (1.02%), and ribose (0.38%), whereas IH-L was mainly composed of D-glucose (58.15%) and D-galactose (23.19%). In the zebrafish oxidative injury model, IH-F showed antioxidant protection rates of 7.19%, 27.50%, and 41.70% at 250, 500, and 1000 μg/mL, respectively, while IH-L showed protection rates of 26.46%, 28.33%, and 43.59% at 25, 50, and 100 μg/mL, respectively. In the CuSO4-induced inflammation model, the highest anti-inflammatory protection rates of IH-F and IH-L were 27.82% and 47.72%, respectively. Both polysaccharides promoted the growth of *Saccharomyces cerevisiae* and mildly inhibited A549 cell proliferation in vitro. These findings suggest that *I. hispidus* polysaccharides may have potential as functional ingredients, although further structural and mechanistic studies are required.

## 1. Introduction

*Inonotus hispidus* is a traditional medicinal mushroom that is widely distributed in Southeast Asian countries [1]. Many bioactive compounds, including polyphenols, triterpenoids, fatty acids, and polysaccharides, have attracted attention because of their pharmacological properties [2,3]. Previous studies have shown that compounds obtained from the fruiting bodies or cultured mycelia of *I. hispidus* possess antioxidant, anti-inflammatory, immunomodulatory, metabolic regulatory, and other biological activities [2,3,4,5,6]. Among these components, polysaccharides are important primary bioactive macromolecules of medicinal fungi, and their monosaccharide composition, molecular weight, chain conformation, and branching patterns are closely associated with their biological activities [7,8,9,10]. Therefore, comparing polysaccharides produced under different cultivation modes is important for understanding how cultivation strategy affects polysaccharide composition and potential function. Broader studies on medicinal mushrooms also support the diversity of their bioactive constituents and biological effects [11].

Previous genomic and metabolomic studies have demonstrated that *I. hispidus* has the genetic and metabolic capacity to produce diverse bioactive compounds. For example, the reported genome of *I. hispidus* is approximately 35.68 Mbp and contains multiple genes associated with secondary metabolite biosynthesis [12,13]. In addition, metabolite profiles vary during mycelial growth and fruiting body development, indicating that growth stage and cultivation conditions can influence the accumulation of bioactive compounds [14]. However, compared with secondary metabolites, less attention has been paid to how cultivation mode affects the primary polysaccharide fractions of *I. hispidus*.

Because wild *I. hispidus* resources are limited, artificial fruiting body cultivation and submerged fermentation are practical approaches for producing fungal polysaccharides. Previous studies have reported polysaccharides from solid-state fermentation substrates, or exopolysaccharides of *I. hispidus* [5,6], and recent work has further examined structural and biological properties of *I. hispidus* polysaccharides [15,16]. Nevertheless, direct comparison of polysaccharides obtained from fruiting bodies and submerged fermentation broth of the same strain under a unified experimental framework remains limited. Such comparison is useful for distinguishing cultivation-dependent differences in polysaccharide composition and preliminary biological responses.

Therefore, this study aimed to compare the physicochemical characteristics and preliminary biological activities of *I. hispidus* polysaccharides obtained from two cultivation modes. The strain LQ-1 was isolated from wild fruiting bodies and identified based on internal transcribed spacer (ITS) sequence comparison and morphological characteristics. Polysaccharides were extracted from artificially cultivated fruiting bodies and submerged fermentation broth. SEM, monosaccharide composition analysis, thermal stability analysis, zebrafish antioxidant and anti-inflammatory assays, A549 cell proliferation assay, and *S. cerevisiae* growth assay were used to compare the two polysaccharide fractions. This work provides a preliminary basis for understanding how cultivation strategy influences *I. hispidus* polysaccharide composition and biological responses.

## 2. Results

### 2.1. Morphological and Molecular Identification

**Figure 1:**
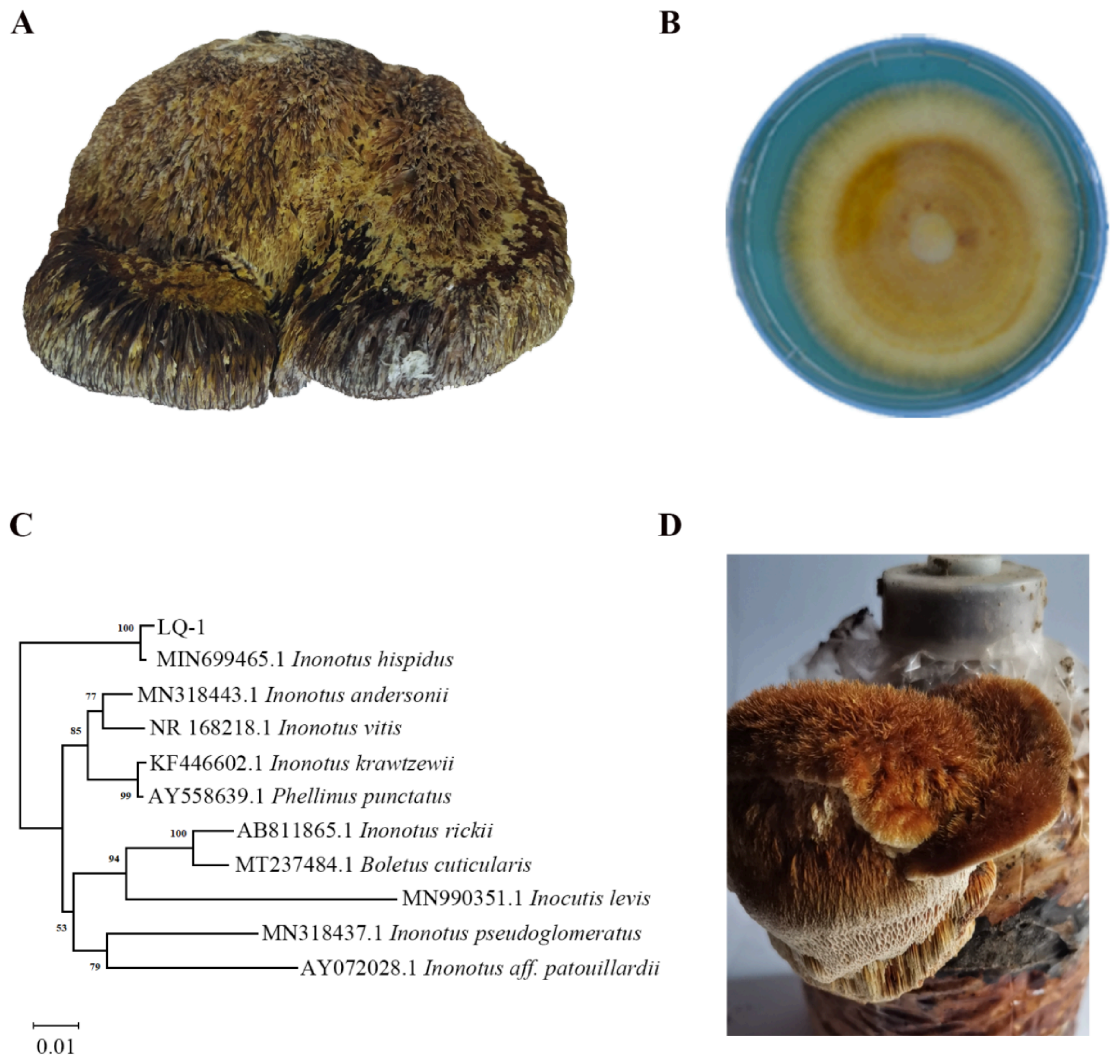
Isolation, phenotypic determination, and phylogenetic tree analysis of *Inonotus hispidus* strains. Wild *I. hispidus* fruiting body (**A**); plate culture photos (**B**); phylogenetic tree (**C**); and photos of solid culture fruiting bodies (**D**). Numbers at the branches indicate bootstrap values.

In the present study, wild fruiting bodies of *I. hispidus* were collected from a *Morus alba* L. tree in Linqing, Shandong Province, China, and strain LQ-1 was isolated from the fresh fruiting bodies (Figure 1A). *Morus alba* L. served only as the host/source plant and was not subjected to sequencing or genetic analysis. The morphological characteristics of LQ-1 are shown in Figure 1B. The ITS sequence obtained from fungal isolate LQ-1 was queried against the NCBI database, and a phylogenetic tree was constructed using the neighbor-joining method with similar sequences retrieved from the NCBI database. As shown in Figure 1C, strain LQ-1 and *I. hispidus* (MIN699465.1) clustered in the same clade with 100% bootstrap support. Based on morphological features and ITS sequence comparison, strain LQ-1 was identified as *I. hispidus*.

The isolate that could produce a fruiting body was identified by performing a fruiting body induction test using strain LQ-1. Environmental conditions such as temperature, nutrients, gas exchange, humidity, and lighting are crucial for the induction of fruiting bodies [17]. The early stage of vegetative mycelium development appeared white in darkness and immediately became yellow after light exposure. Under specific growth conditions, the strain LQ-1 produced large, nutritionally rich sclerotia. After indirect light exposure for 30 days, the medium surface was covered with brown sclerotia with a size of over 0.5 cm. The sclerotium-containing medium was then subjected to fruiting body induction, which was achieved by exposing it to ambient light and exchanging air, while allowing for natural temperature fluctuations between night and day. After 3–4 weeks, the fruiting bodies gradually developed and underwent a change in size, shape, and color. Initially, the fruiting bodies were light yellow and smooth, but upon maturation, they turned yellow to brown or black and acquired a distinctive horseshoe or hemisphere shape (Figure 1D). A large amount of brown coarse hairs was observed on the surface of the fruiting body (Figure 1D). Based on the successful fruiting body initiation of isolate LQ-1 under controlled conditions, the sample was confirmed to be *I. hispidus*.

### 2.2. Extraction of Polysaccharide from I. hispidus Fruiting Body

**Figure 2:**
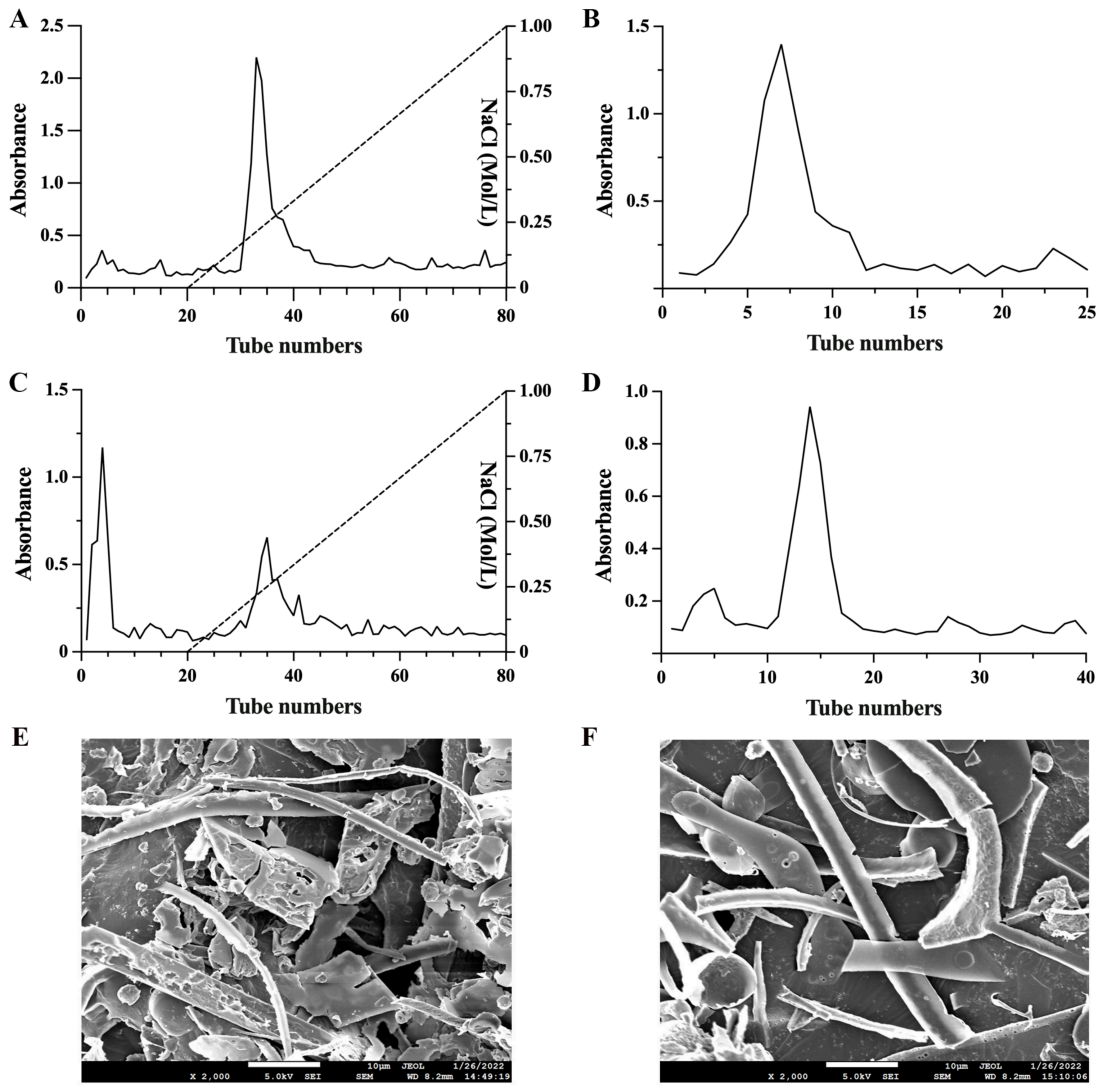
Purification and scanning electron micrographs. Elution profiles of polysaccharide IH-F on a DEAE-52 column (**A**) and a Sephadex G-100 column (**B**). Elution profiles of polysaccharide IH-L on a DEAE-52 column (**C**) and a Sephadex G-100 column (**D**). Scanning electron micrographs of IH-F (**E**) and IH-L (**F**).

Polysaccharides in *I. hispidus* are among the most common active compounds [5]. The extraction of polysaccharide from *I. hispidus* fruiting body involves several stages, including hot water extraction, precipitation using a 95% ethanol solution, activated carbon adsorption for decoloration, and deproteinization with Sevag reagent [18,19]. In the present study, the resulting crude extract had a yield of 1.32%. The obtained crude polysaccharide was purified using an ion-exchange column DEAE-52 and eluted using distilled water and gradient concentrations of NaCl solutions. After isolating one major polysaccharide peak and freeze-drying (Figure 2A), the fraction was subjected to further purification on a Sephadex G-100 column. Only one peak was observed in the elution curve of IH-F (Figure 2B), indicating the high homogeneity of the polysaccharide.

### 2.3. Extraction of a Soluble Polysaccharide from the Fermented Supernatants of I. hispidus LQ-1

Batch fermentation is effective for the mass production of polysaccharides at industrial scale [6]. A polysaccharide was isolated from the supernatants of *I. hispidus* LQ-1 batch fermentation by separating the obtained crude extract through an ion-exchange column DEAE-52 followed by freeze-drying (Figure 2C). The polysaccharide component of the second peak was selected as the main object of study. The purified fraction was subjected to further purification in a Sephadex G-100 column, resulting in only one peak in the elution curve of IH-L (Figure 2D), indicating a high degree of homogeneity.

### 2.4. Surface Characterization of IH-F and IH-L

The composition of fungal polysaccharides directly affects the microstructure of the polysaccharides [20,21,22]. SEM is a powerful imaging technique. The scanning electron microscopy images illustrate the surface morphologies of polysaccharides IH-F and IH-L (Figure 2E,F), respectively. SEM images show that the polysaccharide IH-F is piled up in flakes or debris, and some polysaccharide parts are rod-shaped with a width range of 1–10 μm. Polysaccharide IH-L is flaky or rod-shaped, and the surface of IH-L is smooth. Some flaky polysaccharides have a porous structure. Therefore, IH-F and IH-L, which were produced under different culture modes, had different surface structures and morphologies.

### 2.5. Compositional Analysis of IH-F and IH-L

**Figure 3:**
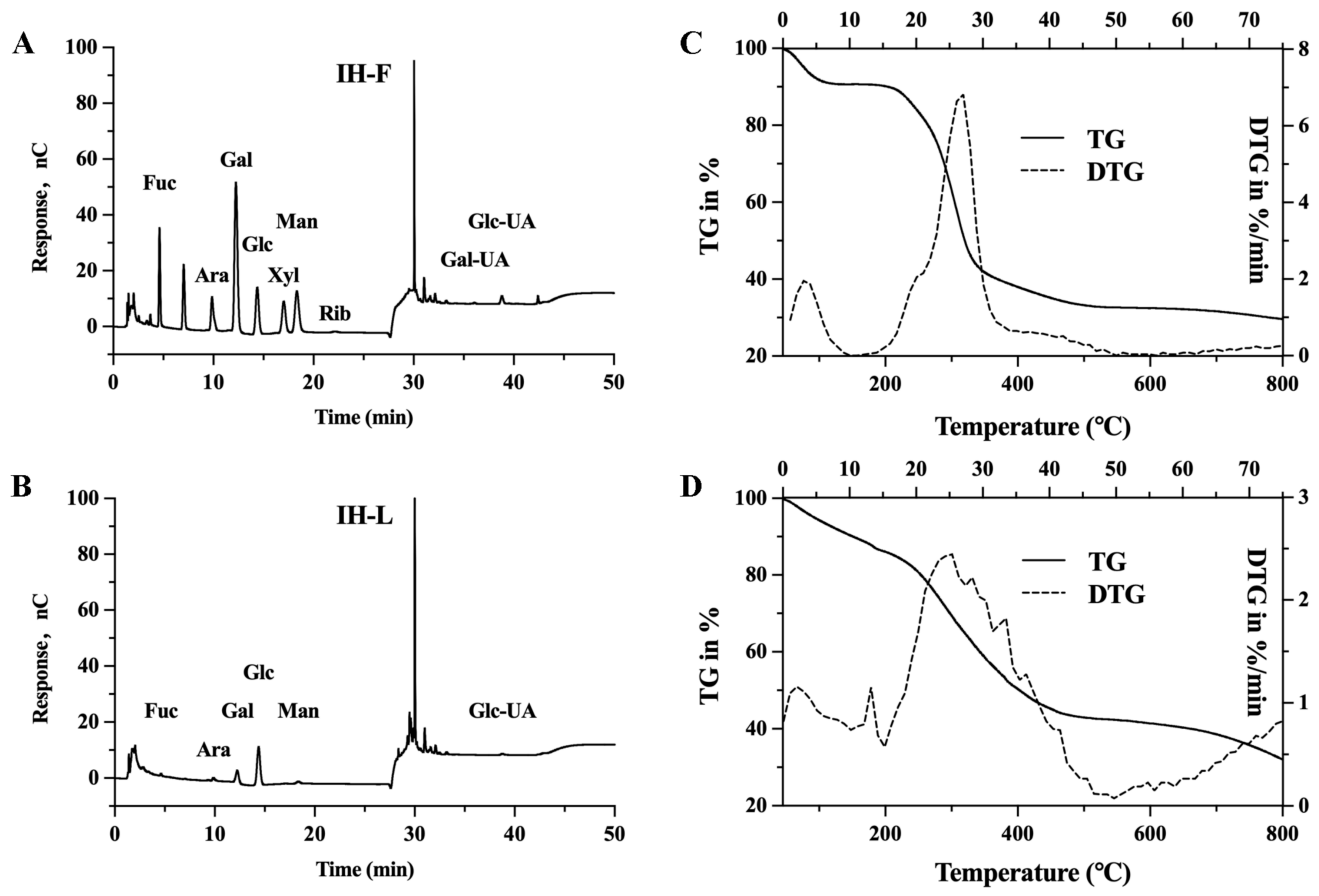
Structural analysis of polysaccharides IH-F and IH-L. Monosaccharide components of IH-F (**A**) and IH-L (**B**). Thermogravimetric analysis of IH-F (**C**) and IH-L (**D**). TG, thermogravimetry; DTG, derivative thermogravimetry.

Single elution peaks were observed for both IH-F and IH-L after purification, suggesting that the collected fractions were relatively homogeneous under the chromatographic conditions used (Figure 2B,D). According to the monosaccharide standards (Figure S1), IH-F was composed of D-galactose (36.07%), L-fucose (17.22%), D-arabinose (9.43%), D-glucose (9.78%), D-xylose (7.29%), D-mannose (15.48%), ribose (0.38%), glucuronic acid (3.32%), and D-galacturonic acid (1.02%) by mass (Figure 3A, Table 1). By contrast, IH-L was mainly composed of D-glucose (58.15%) and D-galactose (23.19%), with smaller proportions of L-fucose (2.77%), D-arabinose (5.85%), D-mannose (4.78%), and glucuronic acid (5.26%) (Figure 3B, Table 1). These results indicate that IH-F and IH-L are acidic heteropolysaccharide fractions and that their monosaccharide compositions differ markedly between the two cultivation modes.

### 2.6. TG of IH-F and IH-L

The TG (%) and derivative TG (DTG, %/min) curves provide the decomposition rate and are helpful for the accurate evaluation of the mass loss steps [20]. The thermal degradation behavior of IH-F was investigated using TG and DTG (Figure 3C). The results showed three distinct stages of degradation. The first stage at 45–110 °C is associated with the evaporation of free water [23], resulting in a mass loss of 8.9%. The second stage at 230–370 °C is the primary degradation stage, and it can be attributed to the degradation of polysaccharide chains [20]. The maximum degradation rate was observed at 310 °C, and 47% of the initial mass was lost during this stage. The third stage at 400–800 °C involved a low mass loss of 3%.

The curves of IH-L featured four main loss steps. Figure 3D shows the TG and DTG curves of IH-L for analysis. The first stage at 45–110 °C led to a mass loss of approximately 7.8% due to the evaporation of free water. The highest mass loss rate was observed at 80 °C. The second stage occurred between 150 and 200 °C, where IH-L exhibited a slow mass loss of 4%. The maximum decomposition rate was observed at 180 °C. The third stage at 250–400 °C was the main degradation stage, which led to a mass loss of approximately 30.4%. This step was related to the sugar chain degradation, and the maximum decomposition rate was recorded at 300 °C. The fourth stage was observed from 400 °C to 800 °C, where the sample exhibited a mass loss of 3%. The weight loss of IH-F (47%) at 230–370 °C was higher than that of IH-L (30.6%) at 250–470 °C. The two major mass loss processes can be attributed to dehydration and decarboxylation, indicating that the thermal stability of IH-F was lower than that of IH-L.

### 2.7. Antioxidant Effects of IH-F and IH-L in Metronidazole-Induced Zebrafish Embryos

**Figure 4:**
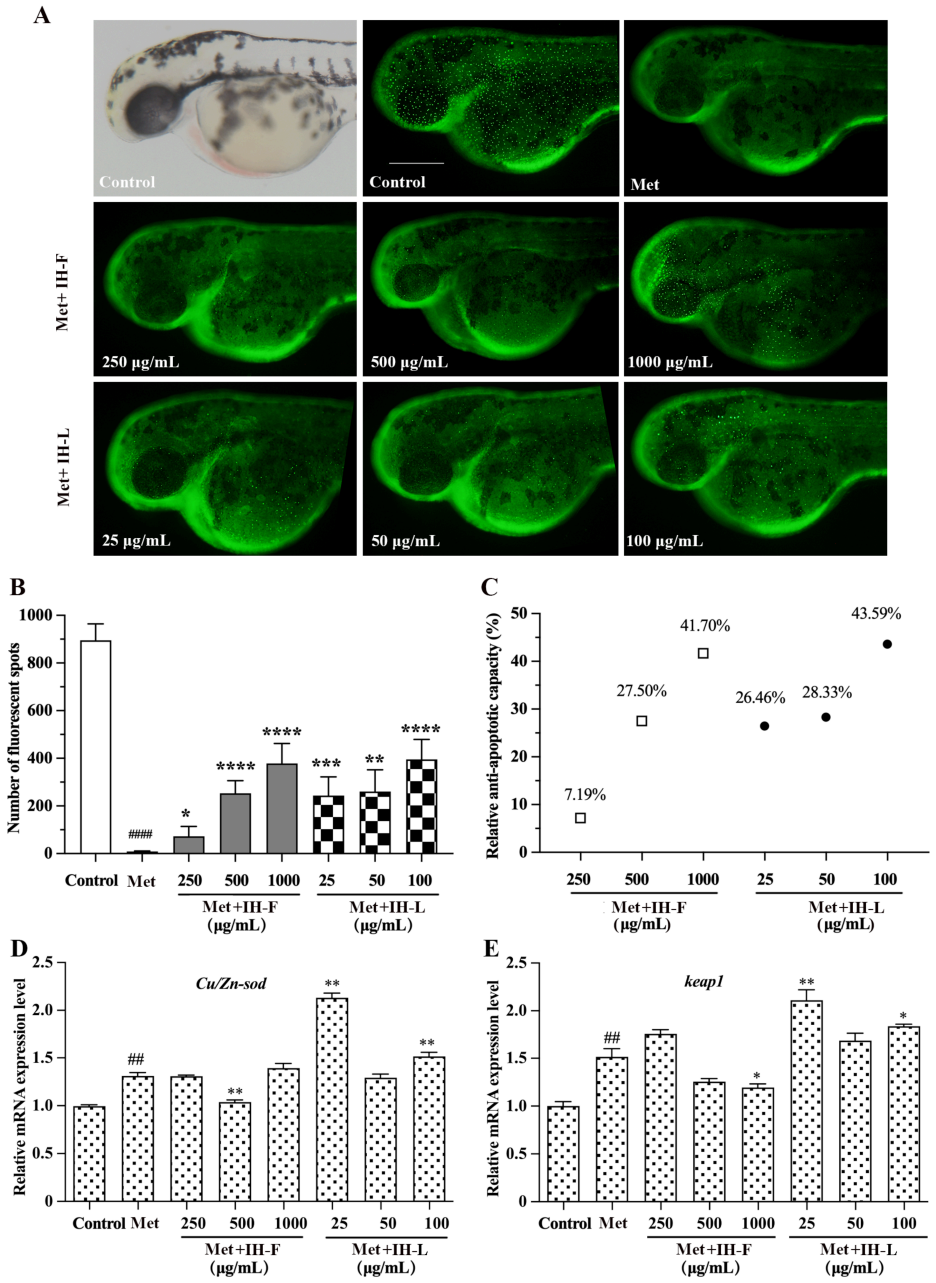
Antioxidant analysis of IH-F and IH-L in a zebrafish model. Fluorescent images (**A**) and calculated total cell number/protection rate (**B**,**C**). The mRNA expression levels of Cu/Zn-sod and keap1 measured by qPCR in zebrafish embryos at 24 hpf after Met exposure (**D**,**E**). Data are expressed as the mean ± SD from three independent biological replicates. ## *p* < 0.01 and #### *p* < 0.0001 versus the blank group; * *p* < 0.05, ** *p* < 0.01, *** *p* < 0.001, and **** *p* < 0.0001 versus the Met group. NTR-GFP, nitroreductase-green fluorescent protein; Met, metronidazole; hpf, hours post-fertilization; qPCR, quantitative PCR. Squares and circles/dots in (**C**) denote IH-F and IH-L groups, respectively. versus the Met group.

In the Met-treated model group, NTR-GFP fluorescence signals were markedly reduced compared with the blank group (*p* < 0.0001; Figure 4A–C) [24]. Treatment with IH-F at 250, 500, and 1000 μg/mL reduced the Met-induced loss of NTR-GFP-positive cells, with relative antioxidant protection rates of 7.19%, 27.50%, and 41.70%, respectively (Figure 4B,C). These results indicate that IH-F attenuated Met-induced oxidative injury in a concentration-dependent manner under the tested conditions.

IH-L also reduced Met-induced fluorescence loss in zebrafish embryos. The relative antioxidant protection rates of IH-L were 26.46%, 28.33%, and 43.59% at 25, 50, and 100 μg/mL, respectively (Figure 4B,C). Compared with IH-F, IH-L achieved comparable antioxidant protection at the lower tested concentrations.

The expression levels of antioxidant-related genes were further examined by RT-qPCR. IH-F significantly reduced keap1 expression (*p* < 0.05) but did not significantly increase Cu/Zn-sod expression (Figure 4D,E). IH-L significantly increased Cu/Zn-sod expression (*p* < 0.01), while keap1 expression was also increased under the tested conditions (Figure 4D,E). These qPCR results suggest that IH-F and IH-L differentially affected antioxidant-related gene expression in zebrafish embryos; however, further oxidative stress markers and pathway analyses are needed to clarify the underlying mechanisms.

### 2.8. Anti-Inflammatory Effects of Polysaccharides IH-F and IH-L

**Figure 5:**
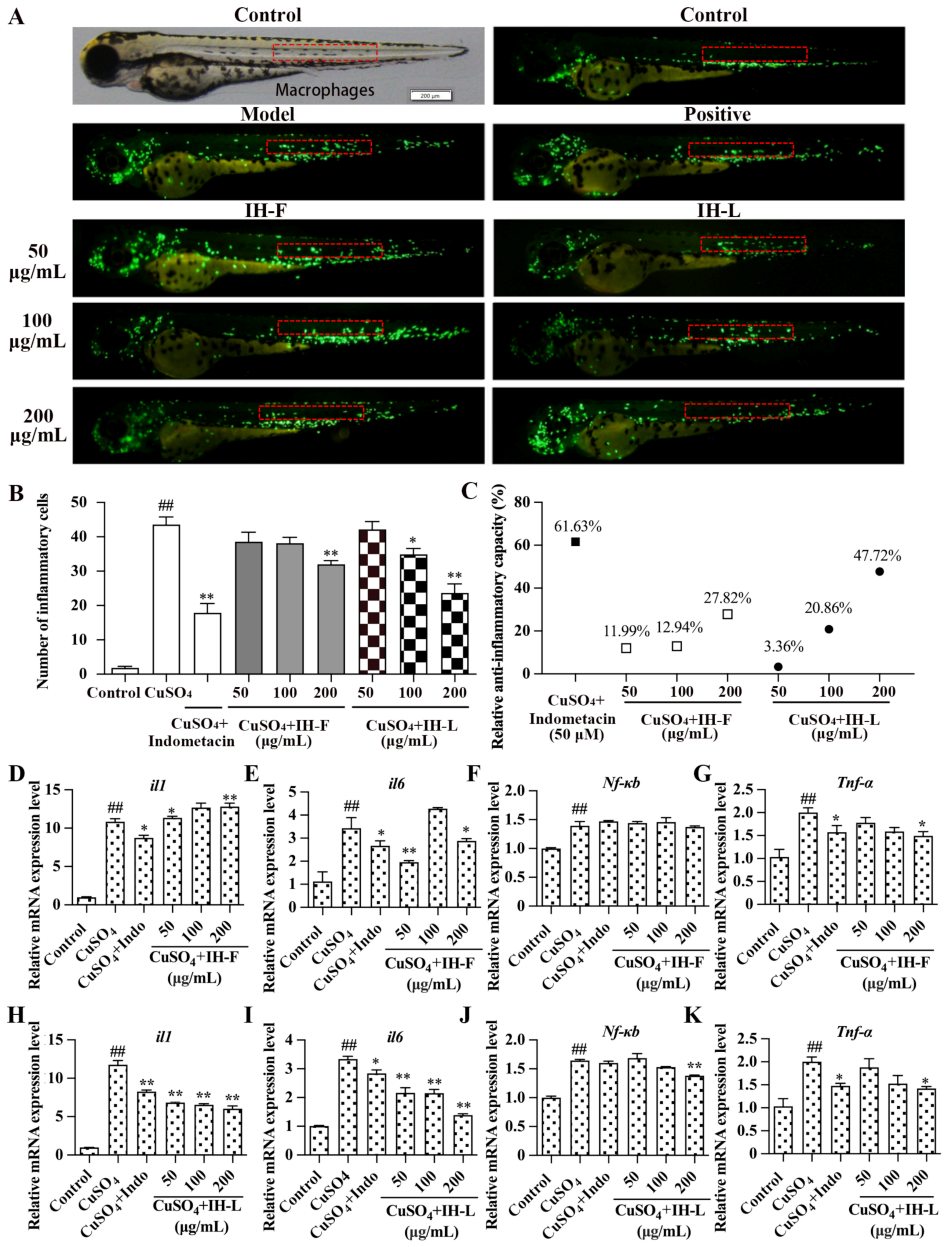
Anti-inflammatory analysis of IH-F and IH-L in a zebrafish model. Fluorescent images (**A**) and calculated total cell number/protection rate (**B**,**C**). The mRNA expression levels of il1, il6, tnf-α, and NF-κB were measured by qPCR in zebrafish embryos after CuSO4 exposure (**D**–**K**). Data are expressed as the mean ± SD from three independent biological replicates. ## *p* < 0.01 versus the blank group; * *p* < 0.05 and ** *p* < 0.01 versus the CuSO4 model group. CuSO4, copper sulfate; qPCR, quantitative PCR; NF-κB, nuclear factor kappa B. Squares and circles/dots in (**C**) denote IH-F and IH-L groups, respectively.

CuSO4 exposure induced macrophage accumulation around the neuromast area in zebrafish larvae (Figure 5A, model group) [25]. Indomethacin reduced macrophage accumulation, confirming the responsiveness of the model (Figure 5A,B). At 50, 100, and 200 μg/mL, IH-F showed relative anti-inflammatory protection rates of 11.99%, 12.94%, and 27.82%, respectively (Figure 5C). IH-L showed relative anti-inflammatory protection rates of 3.36%, 20.86%, and 47.72% at 50, 100, and 200 μg/mL, respectively (Figure 5C). Thus, IH-L produced a higher protection rate than IH-F at the highest tested concentration.

The expression of inflammation-related genes was also measured by RT-qPCR. In the IH-F-treated groups, il6 and tnf-α expression decreased significantly at selected concentrations, whereas il1 and NF-κB showed concentration-dependent or non-significant responses (Figure 5D–G). In the IH-L-treated groups, il1, il6, NF-κB, and tnf-α expression were significantly reduced at one or more tested concentrations (Figure 5H–K). These results suggest that IH-F and IH-L may attenuate CuSO4-induced inflammatory responses by modulating inflammatory gene expression, although protein-level validation and pathway analyses are still required.

### 2.9. Effects of Polysaccharides IH-F and IH-L on A549 Cell Proliferation In Vitro

The effects of IH-F and IH-L on A549 cell proliferation were evaluated using the CCK-8 assay. A549 cells were treated with IH-F or IH-L at 125, 250, 500, 1000, and 2000 μg/mL for 24 h. Both polysaccharide fractions showed mild inhibitory effects on A549 cell proliferation (Figure S2). Because only cell proliferation was assessed in a single cell line, these results should be interpreted as preliminary observations rather than evidence of a complete antitumor mechanism.

### 2.10. Yeast Growth-Promoting Activity of IH-F and IH-L

**Figure 6:**
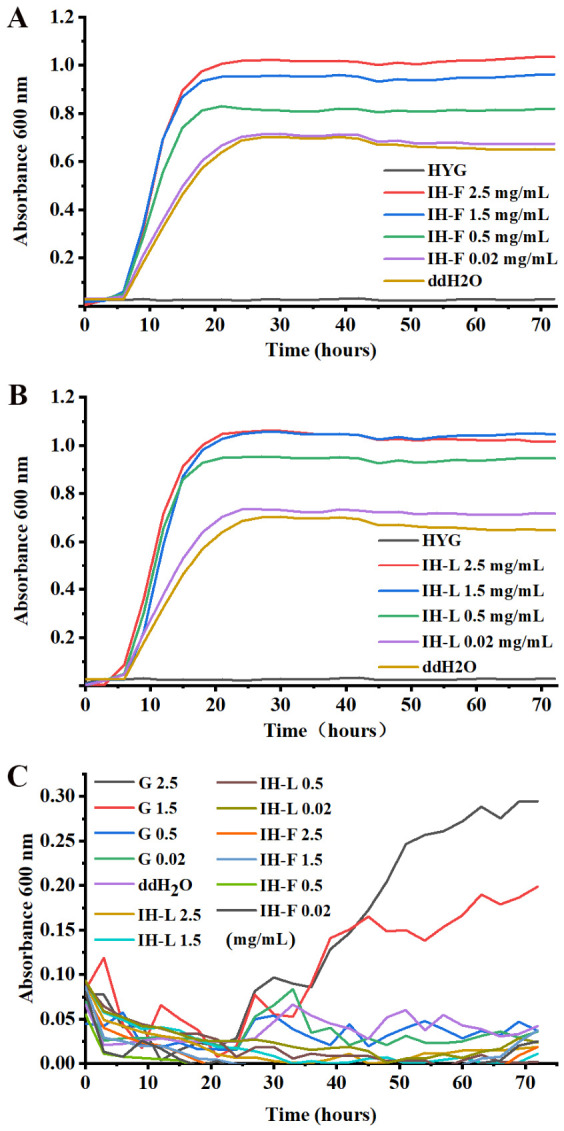
Growth curves of *S. cerevisiae* in malt extract medium (**A**,**B**) and YNB medium (**C**).

The growth-promoting effects of IH-F and IH-L were evaluated using *S. cerevisiae* as a model microorganism. In malt extract medium, both IH-F and IH-L promoted *S. cerevisiae* growth in a concentration-dependent manner (Figure 6A,B). However, in the YNB medium without other carbon sources, neither IH-F nor IH-L supported growth comparable to that observed in the glucose-positive control (Figure 6C). These results indicate that IH-F and IH-L may act as yeast growth-promoting factors under nutrient-containing conditions, but additional tests using probiotic bacteria such as *Lactobacillus* spp. and *Bifidobacterium* spp. are needed before strong prebiotic claims can be made. Similar yeast-based utilization models have been used to evaluate carbohydrate metabolism and yeast growth [26,27].

Polysaccharides not only serve as a source of carbon for microorganisms to grow but also play crucial roles in various biochemical processes [28]. In the present study, to further test whether the rapid growth of *S. cerevisiae* in polysaccharide-treated medium was caused by IH-F and IH-L, we analyzed the growth curve variations using Bioscreen-C in the YNB medium. Cultures in the YNB medium supplemented with different concentrations of glucose were used as positive controls. The YNB medium without a carbon source was used as the blank control. Polysaccharides were added to the YNB medium at varying concentrations (0.02, 0.5, 1.5, and 2.5 mg/mL) to form the sample groups. Figure 6C shows that *S. cerevisiae* growth increased as the glucose concentration increased. Statistical analysis indicated no significant differences between the blank control group without a carbon source and groups treated with varying concentrations of polysaccharides (Figure 6C). Therefore, IH-F and IH-L alone, as carbon sources, could not promote the growth of *S. cerevisiae* at the tested levels. However, in malt extract medium, IH-F and IH-L promoted the growth of *S. cerevisiae* and may be used as yeast growth-promoting factors.

## 3. Discussion

The present study demonstrates that cultivation strategy is an important factor influencing the physicochemical characteristics and preliminary biological activities of polysaccharides from *I. hispidus*. Previous studies have reported antioxidant or protective activities of polysaccharides obtained from *I. hispidus* solid-state fermentation substrates and exopolysaccharides [5,6]. Different from those reports, the present work directly compared polysaccharides obtained from fruiting bodies and submerged fermentation broth of the same strain, allowing cultivation-dependent differences to be evaluated under a unified experimental framework. The results showed that IH-F and IH-L differed in surface morphology, monosaccharide composition, and thermal degradation behavior, suggesting that solid-state fruiting body cultivation and submerged fermentation direct *I. hispidus* toward distinct polysaccharide outputs.

The marked difference in monosaccharide composition may partly explain the distinct biological responses observed in this study. IH-F contained a broader monosaccharide spectrum and relatively high proportions of D-galactose, L-fucose, and D-mannose, whereas IH-L was dominated by D-glucose and contained glucuronic acid. Previous studies on medicinal mushroom polysaccharides have shown that monosaccharide composition, uronic acid content, molecular weight, glycosidic linkage type, and chain conformation can all influence antioxidant, immunomodulatory, and anti-inflammatory activities [7,8,9,10]. In the present study, IH-L produced comparable antioxidant protection at lower concentrations and showed a higher anti-inflammatory protection rate at 200 μg/mL than IH-F. These results suggest that submerged fermentation may produce polysaccharide fractions with different biological responsiveness, although the specific structure–activity relationship remains to be clarified. Similar relationships between polysaccharide composition and biological activity have been reported in other polysaccharide systems [29,30,31].

The zebrafish antioxidant and anti-inflammatory assays provide useful preliminary biological evidence, but the mechanistic interpretation should remain cautious. The antioxidant data were based on fluorescence imaging and the expression of Cu/Zn-sod and keap1, while the anti-inflammatory data were based on macrophage migration and inflammatory gene expression. These indicators suggest that IH-F and IH-L can modulate oxidative injury and inflammatory responses, but they do not fully establish the underlying signaling pathways. Future studies should measure additional oxidative stress markers, such as ROS, MDA, CAT, GPx, and SOD activity, and should validate Nrf2/Keap1, NF-κB, and MAPK signaling at the protein level. Related zebrafish and polysaccharide studies also support the need to evaluate oxidative stress and inflammatory pathways using multiple markers [32,33,34].

The effects on A549 cells should also be interpreted conservatively. The present study only evaluated cell proliferation in one human lung adenocarcinoma epithelial cell line, and apoptosis, cell-cycle distribution, migration, invasion, and related molecular pathways were not examined. Therefore, the A549 results indicate only a mild preliminary inhibitory effect on cell proliferation and should not be considered sufficient evidence for an antitumor mechanism. Previous polysaccharide studies on A549 cells have similarly emphasized the need for mechanistic validation beyond proliferation assays [35].

The *S. cerevisiae* growth assay further showed that IH-F and IH-L promoted yeast growth in malt extract medium but did not serve as an efficient sole carbon source in the YNB medium. Thus, these polysaccharides may function as yeast growth-promoting factors under nutrient-containing conditions rather than as independently utilizable carbon sources under the tested conditions. Because only *S. cerevisiae* was tested, further validation using representative probiotic bacteria, including *Lactobacillus* spp. and *Bifidobacterium* spp., is required before making strong prebiotic claims. Previous studies on polysaccharide-related prebiotic, immunomodulatory, and microbiota-regulating effects also indicate that broader microbial and in vivo validation is needed [36,37,38,39].

This study has several limitations. Molecular weight distribution, FTIR spectra, NMR analysis, glycosidic linkage determination, branching patterns, and conformational characteristics were not determined; therefore, a complete structure–activity relationship cannot yet be established. These structural analyses are important because fungal polysaccharide bioactivities are strongly affected by molecular weight, monosaccharide sequence, linkage pattern, branching degree, and chain conformation. Accordingly, the present results should be regarded as a comparative preliminary evaluation of polysaccharides from two cultivation modes rather than a complete structural or mechanistic characterization. Previous immunomodulatory studies of bioactive polysaccharide complexes further support the need for detailed mechanistic characterization [40].

Overall, the data indicate that fruiting body cultivation and submerged fermentation produce *I. hispidus* polysaccharide fractions with distinct compositional and biological profiles. The stronger antioxidant and anti-inflammatory responses observed for IH-L at lower or comparable concentrations may be related to its cultivation-dependent composition and physicochemical properties. However, additional structural characterization and mechanistic validation are necessary to confirm this interpretation and to evaluate the potential application of *I. hispidus* polysaccharides as functional ingredients.

## 4. Materials and Methods

### 4.1. Reagents and Chemicals

Sephadex G-100 and Cellulose DEAE-52 were obtained from Beijing Solarbio Science & Technology Co., Ltd. (Beijing, China) and GE Healthcare (Chicago, IL, USA), respectively. Standard monosaccharides were obtained from Shanghai Yuanye Bio-Technology Co., Ltd. (Shanghai, China). All analytical-grade biochemical reagents used in this study were procured from Sangon Biotech Co., Ltd. (Shanghai, China).

### 4.2. Strains and Culture Conditions

Wild fruiting bodies of *I. hispidus* were collected from a *Morus alba* L. tree in Linqing, Shandong Province, China. *Morus alba* L. served only as the host/source plant in this study and was not subjected to sequencing or genetic analysis. Following isolation from the fresh fruiting bodies, the strain was cultured on potato dextrose (PD) agar at 30 °C. The fruiting body formation of the isolated strain in pure culture was attempted using a mulberry branch-based medium containing 75% mulberry branches, 4% cottonseed shells, 4% rice husk, 4% bran, 10% corn flour, 1% gypsum, and 2% glucose. The medium composition, moisture level (70%), temperature, humidity, gas exchange, and light conditions were selected according to previous mushroom cultivation reports and preliminary cultivation experience in our laboratory. Polypropylene bags (15 cm × 32 cm) were used for fruiting body production. The isolate was inoculated into fruiting media using 20 mL of liquid inoculum. Following lightless incubation at 26 °C for at least 20 days, the fully colonized media were moved to a temperature-controlled (28 °C) fruiting room with indirect light. Thirty days later, substrate bags were cut with perforation sizes of 70 mm (width) and 10 mm (height), and fruiting bodies were formed under 90% humidity after 21–28 days with medium light.

The *S. cerevisiae* strain was selected for the yeast growth-promoting assay and cultured in a malt extract medium at 30 °C. *I. hispidus* was cultured in a PD liquid medium at 30 °C for 24 h to achieve mycelial cultivation.

### 4.3. Ultrasonic-Enhanced Water Extraction of Polysaccharides from I. hispidus Fruiting Bodies

The extraction procedure was modified from previously reported hot-water and ultrasonic-assisted extraction methods for fungal polysaccharides. The powder from 50 g of *I. hispidus* fruiting bodies was mixed with 2 L of ddH_2_O and heated at 100 °C for 30 min. Ultrasonic-assisted extraction was then performed at 70 °C and 300 W for 30 min, followed by centrifugation (3500 rpm, 10 min) to obtain the extracted polysaccharide. The concentrated aqueous extract was precipitated with 75% (*v*/*v*) ethanol. The resulting sediment was collected after overnight refrigeration at 4 °C and then subjected to centrifugation (4500 rpm, 10 min). The sediment was washed twice with 95% ethanol to obtain crude polysaccharides, which were then freeze-dried. Activated carbon was used for decolorization, proteins were removed using the Sevag method [19], and the sample was subjected to dialysis for 48 h. The purified polysaccharides were then freeze-dried for further use.

### 4.4. Batch Fermentation

The *I. hispidus* LQ-1 strain was utilized to produce extracellular polysaccharides using a sterilized PD medium prepared in our laboratory from analytical-grade reagents. In a 50 L fermenter (BLBIO-50SJ; Shanghai Bailun Bio-technology Co., Ltd., Shanghai, China), 4 L seed liquid was added at 10% inoculation concentration and fermented at 30 °C under aerobic conditions for 120 h. During the polysaccharide extraction and purification stage, the fermented solution was passed through a filter and centrifuged at 3500 rpm for 10 min. The resulting supernatants were collected and concentrated to an appropriate volume by using a rotary evaporator. The polysaccharides were precipitated by adding 75% ethanol to the solution. The resulting solution was stored at 4 °C for 12 h.

### 4.5. Purification of Polysaccharides

The extracted polysaccharide was purified using a DEAE-52 column (2.6 cm × 20 cm). The elution process involved the use of distilled water for the first 20 tubes and a linear gradient of NaCl solution (0–1 M) for 21–80 tubes. The flow rate was set at 7 mL/min, and the eluate (13 mL/tube) was collected automatically and monitored using the phenol–sulfuric acid method at 490 nm [18]. The Bradford method was used to determine residual protein in the eluent. Purified polysaccharide components were obtained, and a major polysaccharide peak was detected. After dialysis for NaCl removal, the freeze-dried sample was further purified on a Sephadex G-100 column (1.6 cm × 60 cm) by using distilled water (0.3 mL/min, 3 mL/tube). The mass of polysaccharide samples purified by the Sephadex G-100 column was about 300 mg each time. The eluate was collected automatically and monitored using the method above. The fraction corresponding to the major peak was then lyophilized and used for further analysis.

### 4.6. SEM Analysis

Surface morphology was examined using a scanning electron microscope (SEM, JSM-7610FPlus; JEOL Ltd., Tokyo, Japan). Before SEM analysis, samples were gold-coated. Magnifications of 1000×–5000× were selected to observe both the overall surface morphology and local microstructural features of the polysaccharide samples.

### 4.7. Monosaccharide Composition

For monosaccharide composition analysis, 5 mg of sample was subjected to hydrolysis using 2 M trifluoroacetic acid. Hydrolysis was carried out in a sealed tube, and the conditions were maintained at 121 °C for 2 h. The sample was then washed several times with methanol, re-dissolved in ddH_2_O, filtered through a microporous filtering film (0.22 μm), and analyzed using high-performance anion-exchange chromatography on a CarboPac PA-20 anion exchange column with a pulsed amperometric detector (PAD) system. The gradient program involved a solvent system of ddH_2_O, 0.2 M NaAc, and 0.1 M NaOH. The resulting data were analyzed using Chromeleon 7.2 CDS (Thermo Fisher Scientific, Waltham, MA, USA).

### 4.8. Thermal Stability Analysis

The thermal properties of the sample were determined via thermogravimetric analysis (TG) using a Mettler Toledo Instruments apparatus (Mettler Toledo, Greifensee, Switzerland). Approximately 2.5 mg of sample was tested under a nitrogen atmosphere. Prior to the test, the temperature was increased from 45 °C to 800 °C at a heating rate of 10 °C/min.

### 4.9. Zebrafish Oxidative Injury Assay

A transgenic zebrafish line called (Krt4:NTR-GFP)cy17 was developed to induce apoptosis specifically in superficial skin cells [24]. This line can be activated conditionally. The killer line showed extensive apoptosis upon exposure to the prodrug metronidazole (Met) during incubation. Met was prepared as a 10 mM stock solution by using double-distilled water and stored at −20 °C. Adult zebrafish samples were kept in a controlled environment and fed twice a day with *Artemia nauplii*. Embryos were obtained naturally from these adult zebrafish. The sample groups were treated with polysaccharides IH-F (250, 500, and 1000 μg/mL) or IH-L (25, 50, and 100 μg/mL) and prodrug Met (5 mM), while the control and blank groups were only treated with Met (5 mM) and fresh fish water, respectively. After 24 h, the number of Krt4:NTR-GFP skin cells was counted and statistically analyzed. The fluorescent images were captured using a fluorescence microscope. The total cell number was calculated using ImageJ software (version 1.53t; National Institutes of Health, Bethesda, MD, USA), and the relative anti-apoptotic capacity was determined using the equation relative anti-apoptotic capacity (%) = (A1 − A2)/(A0 − A2) × 100, where the blank group has a fluorescent signal denoted as A0, while the sample and control groups have fluorescent signals denoted as A1 and A2, respectively. All zebrafish experiments were approved by the Animal Welfare Ethical Committee of the Biological Research Institute at the Shandong Provincial Academy of Sciences (approval No. SWS20230116; 13 January 2023).

### 4.10. Anti-Inflammatory Activities in Zebrafish

The anti-inflammatory properties of the sample were evaluated by inducing an inflammation model in zebrafish by using CuSO4 [25]. Healthy zebrafish larvae were chosen at 24 h post-fertilization and allotted at random to eight-well plates (with 10 larvae per well) at 72 h post-fertilization. IH-F or IH-L polysaccharides (50, 100, and 200 μg/mL) were incubated with the sample groups. The positive group was incubated with indomethacin (50 μM). The model and blank groups were incubated with fresh fish water. All groups were maintained at 28 °C for 2 h after adding polysaccharides or indomethacin. Then, 20 μM CuSO4 was added to the model, positive, and sample groups. After incubation for 1 h, the resulting inflammatory reaction was imaged. All treatments were carried out in triplicate. Fluorescence signals in zebrafish larvae were monitored and captured using an Olympus SZX16 fluorescence microscope (Olympus Corporation, Tokyo, Japan).

### 4.11. RT-qPCR Analysis

TRIzol reagent (Invitrogen, Thermo Fisher Scientific, Waltham, MA, USA) was used to isolate total RNA from 30 zebrafish larvae of each concentration. PrimeScript RT Kit (Takara Bio Inc., Kusatsu, Japan) was then used to synthesize cDNA following the manufacturer’s instructions. Real-time PCR was carried out using the SYBR Green mix (Takara Bio Inc., Kusatsu, Japan) with three technical replicates and three independent biological replicates. The housekeeping gene β-actin was used for normalization, and relative gene expression was calculated using the comparative Ct method. The primers used in this study are listed in Table S1.

### 4.12. Cell Proliferation Assay

The human lung adenocarcinoma epithelial cell line A549, obtained from the American Type Culture Collection (ATCC, Manassas, VA, USA), was used for the cell proliferation assay. Each well of a 96-well plate contained 1 × 10^4^ cells. After culturing at 37 °C for 24 h, the cells were treated with polysaccharide IH-F or IH-L at varying concentrations (0, 125, 250, 500, 1000, and 2000 μg/mL) for another 24 h. The treated cells were then imaged and examined under a microscope. Cell viability was evaluated using the Cell Counting Kit-8 (CCK-8; Dojindo Molecular Technologies, Kumamoto, Japan) assay by adding 10 μL of CCK-8 to each well and incubating for 1 h at 37 °C. The absorbance at 450 nm was measured and compared with untreated cells. The relative inhibition rate (%) was calculated using the formula ([A1 − A2]/[A1 − A0]) × 100, where A0, A1, and A2 represent the absorbance values of the blank, control, and polysaccharide-treated groups, respectively.

### 4.13. Yeast Growth-Promoting Assay

The yeast growth-promoting effects of polysaccharides IH-F and IH-L were evaluated by measuring the growth curves of *S. cerevisiae* in malt extract medium (MEM) and yeast nitrogen base (YNB) medium (13.4 mg/mL), respectively. The culture in the YNB medium supplemented with glucose was used as the positive control. The YNB medium without a carbon source was used as the blank control, and the YNB medium with hygromycin (350 μg/mL) was used as the negative control. Media supplemented with different concentrations (0, 0.02, 0.5, 1.5, and 2.5 mg/mL) of polysaccharides were used as sample groups. The optical density (OD) of *S. cerevisiae* was monitored using Bioscreen-C system (Oy Growth Curves Ab Ltd., Helsinki, Finland) at 600 nm over time [26,27].

### 4.14. Statistical Analysis

All experiments were performed with at least three independent biological replicates unless otherwise stated. Data are presented as the mean ± SD. Statistical analysis was performed using GraphPad Prism 9 (GraphPad Software, San Diego, CA, USA). Differences among multiple groups were analyzed by one-way analysis of variance followed by an appropriate multiple-comparison test. A value of *p* < 0.05 was considered statistically significant.

## 5. Conclusions

In conclusion, two polysaccharide fractions, IH-F and IH-L, were obtained from the fruiting bodies and submerged fermentation broth of *I. hispidus* LQ-1, respectively. The two fractions differed in monosaccharide composition, thermal behavior, and preliminary biological responses. IH-L showed relatively stronger antioxidant and anti-inflammatory effects in zebrafish models at lower or comparable concentrations, while both polysaccharides promoted *S. cerevisiae* growth under nutrient-containing conditions and mildly inhibited A549 cell proliferation. These results indicate that cultivation strategy may influence the composition and preliminary bioactivity of *I. hispidus* polysaccharides. However, detailed structural characterization, including molecular weight distribution, FTIR, NMR, glycosidic linkage analysis, branching patterns, and conformational analysis, as well as further mechanistic studies, are still required before their functional applications can be fully evaluated.

## Figures and Tables

**Table 1:** Monosaccharide composition of IH-F and IH-L.

Peak No.	Fuc	Ara	Rha	Gal	Glc	Xyl	Man	Fru	Rib	Gal-UA	Gul-UA	Glc-UA	Man-UA
IH-F	17.22%	9.43%	0.00%	36.07%	9.78%	7.29%	15.48%	0.00%	0.38%	1.02%	0.00%	3.32%	0.00%
IH-L	2.77%	5.85%	0.00%	23.19%	58.15%	0.00%	4.78%	0.00%	0.00%	0.00%	0.00%	5.26%	0.00%

Abbreviations: Fuc, fucose; Ara, arabinose; Rha, rhamnose; Gal, galactose; Glc, glucose; Xyl, xylose; Man, mannose; Fru, fructose; Rib, ribose; Gal-UA, galacturonic acid; Gul-UA, guluronic acid; Glc-UA, glucuronic acid; Man-UA, mannuronic acid.

## Data Availability

The data presented in this study are available from the corresponding author upon reasonable request.

## Supplementary Materials

The following supporting information can be downloaded at https://www.mdpi.com/article/10.3390/ijms27156724/s1.

## Abbreviations

The following abbreviations are used in this manuscript:

IH-F	Polysaccharide extracted from the fruiting body of *Inonotus hispidus*
IH-L	Polysaccharide extracted from the liquid fermentation broth of *Inonotus hispidus*
SEM	Scanning electron microscopy
TG	Thermogravimetric analysis
DTG	Derivative thermogravimetry
RT-qPCR	Reverse-transcription quantitative PCR
Met	Metronidazole
MEM	Malt extract medium
YNB	Yeast nitrogen base
OD	Optical density
NTR-GFP	Nitroreductase-green fluorescent protein
