# Supplementary material for: Comparison of Biological Activities of Inonotus hispidus Polysaccharides by Solid-State and Submerged Cultures
Source: Int J Mol Sci. 2026 Jul 28;27(15):6724. doi: 10.3390/ijms27156724 (PMC13467579; doi:10.3390/ijms27156724)
Supplement: Supplementary file 1 [file ijms-27-06724-s001.zip › ijms-4384063-supplementary.pdf]

Supplementary Materials

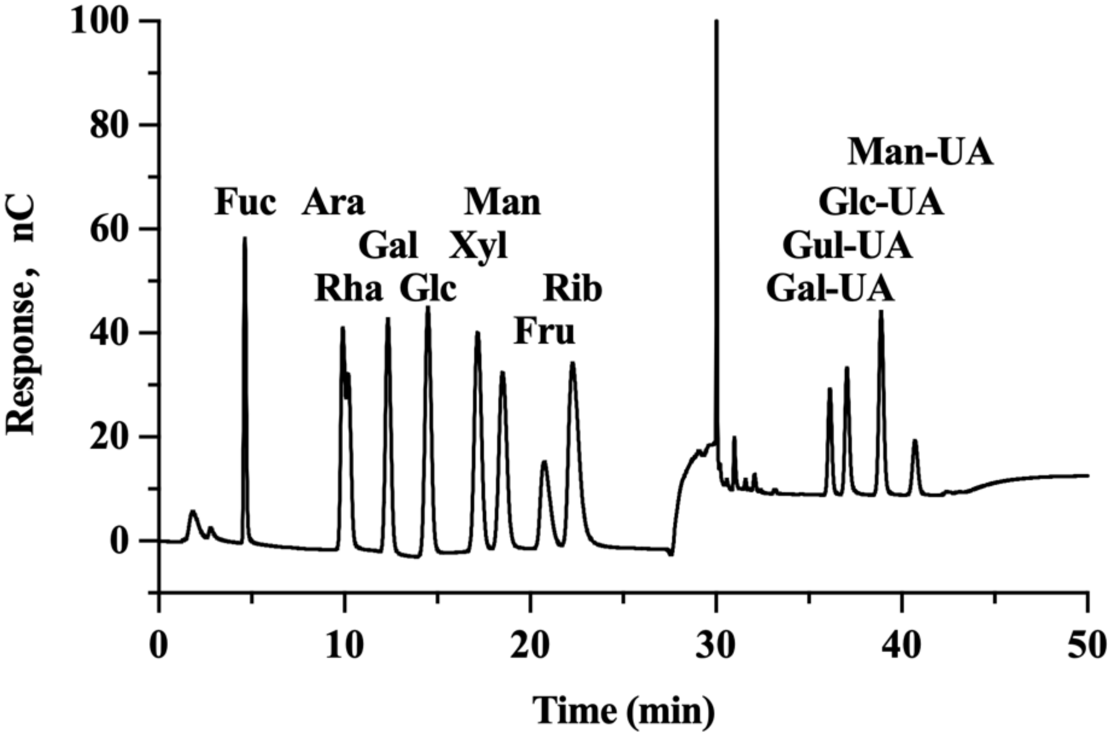

Figure S1. Gel permeation chromatography of the monosaccharide standards.

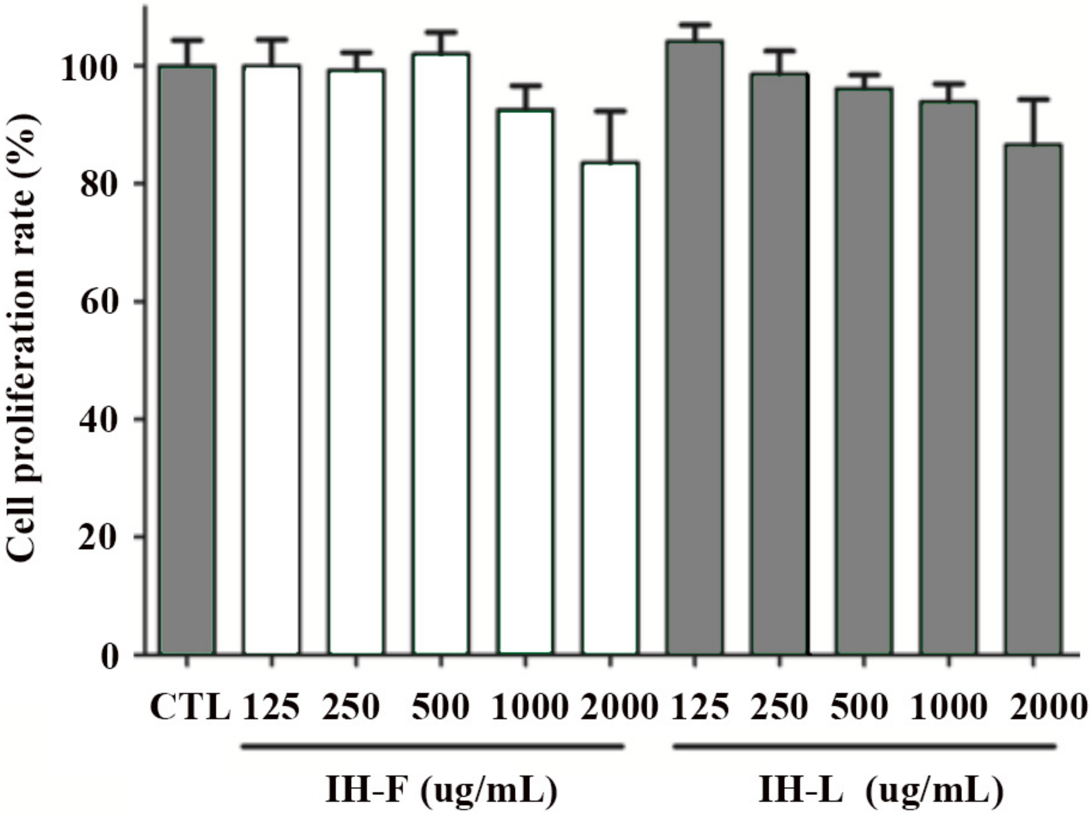

Figure S2. Effects of polysaccharides IH-F and IH-L on A549 cell proliferation in vitro.

Table S1. Primer pairs used in real-time quantitative PCR assay.

Gene	Primer	Primer sequence (5'-3')
β-actin	β-actin-F	ctccggtatgtgcaaagc
	β-actin-R	ccatcactccctgatgtct
il1	il1-F	ctccacatctcgtactcaa
	il1-R	gtccatctccaccatctg
il6	il6-F	cttgattgccgttcagttag
	il6-R	tgaccggagattgtagacac
Tnf-α	Tnf-α-F	ctttaccgctggtgatggtgtcc
	Tnf-α-R	aaagacacctggctgtagacaaa
Nf-κb	Nf-κb-F	acaagacgcaaggagcccag
	Nf-κb-R	aactgtctcttgcacaaagggctca
keap1	keap1-F	acatggagtctcagtctacc
	keap1-R	ggcatatctgttacaagcgt
Mn-sod	Mn-sod-F	tagatgtctgggaacatgcg
	Mn-sod-R	tggctttaacatagtccggtc

References:

15. Liu, X.; Wang, Q.; Wang, J.; Guo, L.; Chu, Y.; Ma, C.; Kang, W. Structural characterization, chain conformation and immunomodulatory activity of a heteropolysaccharide from Inonotus hispidus. *Int. J. Biol. Macromol.* **2024**, *260*, 129187.
16. Zhang, Y.; Zhang, X.; Jin, X.; Li, Z.; Li, L.; Zhu, Y.; Kong, F.; Wang, D. Structural characterization of polysaccharide isolated from Inonotus hispidus and its anti-obesity effect based on regulation of the interleukin-17-mediated inflammatory response. *Int. J. Biol. Macromol.* **2025**, *291*, 138975.
